# β-Sitosterol alleviates the malignant phenotype of hepatocellular carcinoma cells via inhibiting GSK3B expression

**DOI:** 10.1007/s13577-024-01081-y

**Published:** 2024-05-30

**Authors:** Ruoyu Wang, Dan Tang, Longyun Ou, Jiacheng Jiang, Yu-nan Wu, Xuefei Tian

**Affiliations:** 1 Department of Hepatology, The First Hospital of Hunan University of Chinese Medicine, Changsha, 410007 Hunan China; 2 Department of Internal Medicine, College of Integrated Chinese and Western Medicine, Hunan University of Chinese Medicine, Changsha, 410208 Hunan China; 3 Hunan Province University Key Laboratory of Oncology of Tradional Chinese Medicine, Hunan University of Chinese Medicine, Changsha, 410208 Hunan China

**Keywords:** β-Sitosterol, Hepatocellular carcinoma, Glycogen synthase kinase 3 beta, Network pharmacology, Epithelial–mesenchymal transition

## Abstract

To explore the effects of β-Sitosterol upon hepatocellular carcinoma cell proliferation, apoptosis, migration, invasion, and epithelial–mesenchymal transition (EMT), and to investigate the underlying mechanism using network pharmacology. Human hepatocellular carcinoma cell lines (Huh-7 and HCCLM3) were expose to gradient concentrations of β-Sitosterol (5 μg/mL, 10 μg/mL, and 20 μg/mL). Cell viability and proliferation were assessed using MTT, CCK-8, colony formation, and EdU assays.Flow cytometry was employed to evaluate cell cycle and apoptosis. Scratch and Transwell assays were performed, respectively, to detect cell migration and invasion. The levels of apoptosis-associated proteins (BAX, BCL2, and cleaved caspase3) as well as EMT-associated proteins (E-cadherin, N-cadherin, Snail, and Vimentin) were detected in Huh-7 and HCCLM3 cell lines using Western blot analysis. The drug target gene for β-Sitosterol was screened via PubChem and subsequently evaluated for expression in the GSE112790 dataset. In addition, the expression level of glycogen synthase kinase 3 beta (GSK3B) within the Cancer Genome Atlas-Liver Hepatocellular Carcinoma (TCGA-LIHC) database was analyzed, along with its correlation to the survival outcomes of patients with hepatocellular carcinoma. The diagnostic efficiency of GSK3B was assessed by analyzing the ROC curve. Subsequently, Huh-7 and HCCLM3 cell lines were transfected with the overexpression vector of GSK3B and then treated with β-Sitosterol to further validate the association between GSK3B and β-Sitosterol. GSK3B demonstrated a significantly elevated expression in patients with hepatocellular carcinoma, which could predict hepatocellular carcinoma patients’ impaired prognosis based on GEO dataset and TCGA database. GSK3B inhibitor (CHIR-98014) notably inhibited cell proliferation and invasion, promoted cell apoptosis and cell cycle arrest at G0/G1 phase in hepatocellular carcinoma cells. β-Sitosterol treatment further promoted the efffects of GSK3B inhibitor on hepatocellular carcinoma cells. GSK3B overexpression has been found to enhance the proliferative and invasive capabilities of hepatocellular carcinoma cells. Furthermore it has been observed that GSK3B overexpression, it has been obsear can partially reverse the inhibitory effect of β-Sitosterol upon hepatocellular. β-Sitosterol suppressed hepatocellular carcinoma cell proliferation and invasion, and enhanced apoptosis via inhibiting GSK3B expression.

**Supplementary Information:**

The online version contains supplementary material available at 10.1007/s13577-024-01081-y.

## Introduction

Hepatocellular carcinoma is one of the most frequently observed malignancies [1]. In China, individuals aged 50 years and older exhibit a higher susceptiblity to hepatocellular carcinoma [2]. Currently, surgical excision and liver transplantation are considered the primary methods for treating of hepatocellular carcinoma [3]. However, a majortiy of hepatocellular carcinoma cases are diagnosed at an advanced stage when surgical intervention proves ineffective [4], necessitating the utilizaiton of immunotherapy and interventional therapy as essential treatments for advanced and metastatic liver cancers [5, 6]. Nevertheless, the mechanism of immunotherapy and drug resistance remains unknown, and further investigation to evaluate its therapeutic applicability in combination with targeted therapies and other medications.

Recently, natural compounds and traditional Chinese medicine (TCM) have gaining attention for their highly efficient and low-toxicity anti-cancer properties [7]. *Prunus persica* (L.) BATSCH* (Rosaceae)* seeds are well-known in TCM as a traditional medicine (Persicae Semen, Taoren) [8, 9]. Although, frequently used as an ingredient in various prescriptions for treating women’s diseases, its anti-cancer effects have been investigated in recent years [10]. β-Sitosterol is the main compound of Taoren and has been found to possess diverse physiologic activities such as anti-hypercholesterolemia, anti-inflammatory, and antibacterial properties [11]. Moreover, β-Sitosterol’s anti-cancer property has also been revealed within a variety of malignancies including colon carcinoma [12] and breast carcinoma [13], while exhibiting inhibitory effects on hepatocellular carcinoma growth [14]. However, further exploration of the molecular mechanism underlying β-Sitosterol’s anti-cancer effect on hepatocellular carcinoma is still needed.

Network pharmacology has recently emerges as a valuable method that integrates system biology and bioinformatics to elucidate the complex mechanisms of drugs [15]. It provides a theoretical foundation for future emphasis on natural drugs [16]. In a previous study, network pharmacology and bioinformatics approaches were employed to uncover the molecular targets of Fumaria indica in hepatocellular carcinoma therapy [17]. Therefore, it is reasonable to utilize network pharmacology for screening drug target genes of β-Sitosterol in hepatocellular carcinoma. In this study, glycogen synthase kinase 3 beta (GSK3B) was identified as a drug target of β-Sitosterol using network pharmacology. GSK3B is a serine/threonine protein kinase involved in multiple normal and pathologic cell functions such as cell signaling and metabolism [18]. It exhibits high expression levels in various cancers including hepatocellular carcinoma, playing a significant role in regulating tumor proliferation, cell cycle, apoptosis, and chemoresistance [19, 20]. However, the precise mechanism by which β-Sitosterol exerts it effects on hepatocellular carcinoma through modulation of GSK3B remains unclear.

In this study, our aim was to elucidate the biologic functions and potential regulatory mechanism of β-Sitosterol in hepatocellular carcinoma cells. We confirmed that β-Sitosterol suppresses cell proliferation, migration, and invasion in hepatocellular carcinoma. Using network pharmacology, we identified GSK3B as a drug target of β-Sitosterol and verified its regulation of biologic activities by inhibiting GSK3B expression. These findings provide insight into the potential mechanism of β-Sitosterol on hepatocellular carcinoma cells and its clinical applications.

## Materials and methods

### Cell treatment and transfection

Human hepatocellular carcinoma cell lines Huh-7 (CL-0120) and HCCLM3 (CL-0278), and 293 T cells procured from Procell Life Science & Technology Co., Ltd. (Wuhan, China) were cultured in DMEM media added with 10% fetal bovine serum (FBS) and 100 U/mL penicillin/streptomycin in a humidified incubator (37 ℃, 5% CO_2_).

For β-Sitosterol treatment or GSK3B inhibitor (CHIR-98014), β-Sitosterol (Sigma-Aldrich, St. Louis, USA) was first dissolved in DMSO and then diluted with complete culture medium. Huh-7 and HCCLM3 cells were incubated with 5 μg/mL, 10 μg/mL, or 20 μg/mL of β-Sitosterol for 24, 48 or 72 h. CHIR-98014 (MedChem Express, Shanghai, China) was treated to Huh-7 and HCCLM3 cells at 50 nM concentration for 48 h [21].

The GSK3B overexpression plasmid (pLVX-Puro-GSK3B plasmid) and blank pLVX-Puro vector (named vector-NC) were provided by Oribio (Changsha, China). Polymerase chain reaction (PCR) was performed using DNA containing the GSK3B sequence as the template. The amplification products were isolated and purified using 1% agarose gel electrophoresis. The target fragments were retrieved and double digested with XhoI and EcoRI. After further purification, the GSK3B CDS fragment was obtained, and then was sub-cloned into pLVX vector by homologous recombination method using Seamless Cloning Kit (Beyotime, Shanghai, China). Then, the GSK3B pLVX vector and blank pLVX vector were transfected into Huh-7 and HCCLM3 cell lines to a final concentration of 1 μg/ml using Lipofectamine 2000 (Thermo Fisher Scientific, Waltham, USA) according to the manufacturer’s protocols. The primer sequences of the GSK3B pLVX vector construction are shown as follows: Forward: 5′-ATGATGACGATAAAGGATCCATGTCAGGGCGGCCCAGA-3′. Reverse: 5′-CCGGTAGAATTATCTAGATCAGGTGGAGTTGGAAGCTG-3′. The GSK3B pLVX vector reports were provided in the Supplementary Material_1. After transfection for 48 h, the cells were treated with 10 μg/mL β-Sitosterol for another 48 h.

### 3-(4,5-dimethylthiazol-2-yl)-2,5-diphenyltetrazolium bromide (MTT) assay

Huh-7, HCCLM3 and 293 T cell lines were plated onto 96-well plates (5 × 10^3^ cells/well) and cultured in 100 μL of media. All these cell lines were treated with β-Sitosterol at gradient concentrations (5 μg/mL, 10 μg/mL, and 20 μg/mL) for 24, 48, and 72 h, respectively. Next, each well was introduced to 20 μL of MTT (5 mg/mL). After incubation at 37 ℃ for 4 h, each well was added with 100 μL DMSO, followed by a 10 min gentle shaking. A multifunctional microplate reader (PerkinElmer, Shanghai, China) was employed to measure the optical density (OD) at 490 nm.

### Cell counting kit-8 (CCK-8) assay

Huh-7 and HCCLM3 cell lines were seeded onto 96-well plates (5 × 10^3^ cells per well). After GSK3B overexpression plasmid transfection and β-Sitosterol or CHIR-98014 treatment or for 24, 48 and 72 h, 10 μL of CCK-8 solution was added to each well. After 2 h incubation, a multifunctional microplate reader (PerkinElmer) was employed to measure the OD value at 450 nm.

### Colony formation assay

Huh-7 and HCCLM3 cell lines were plated onto six-well plates (1 × 10^3^ cells per well) and incubated overnight. The cells were then treated with β-Sitosterol or CHIR-98014 or 48 h or transfected for 48 h, followed by an incubation for 1–2 weeks with weekly medium refreshed. After the experiment, the culture media was removed. The cells were rinsed in phosphate-buffered saline (PBS), fixed in 4% paraformaldehyde at room temperature (RT) for 20 min, and stained with 0.1% crystal violet for 1 min. Finally, colonies were counted using an optical microscope (Nikon, Tokyo, Japan).

### EdU assay

The cell proliferation detection kit (Guangzhou RiboBio Co., Ltd, Guangzhou, China) was used to detect cell proliferation. In brief, Huh-7 and HCCLM3 cell lines were seeded onto 96-well plates (1 × 10^4^ cells per well) and treated with β-Sitosterol or transfected with plasmids for 48 h. After that, 100 μL EdU media (50 μM) was added to each well for 2 h incubation. After PBS washing, each well was added with 50 μL PBS solution containing 4% paraformaldehyde for 30-min incubation at RT. The fixed solution was then removed. Next, 50 μL glycine solution (2 mg/mL) and 100 μL PBS solution containing 0.5% Triton X-100 were added to each well for 10-min incubation. Afterward, cells were subjected to 30 min staining at RT with 100 μL 1 × Apollo staining reaction solution in the dark. The nucleus was counter-stained using a Hoechst33342 reaction solution. An inverted fluorescence microscope (Olympus, Tokyo, Japan) was employed to observe EdU-positive cells (red) [22].

### Flow cytometry

A PI staining kit (Beyotime) was employed to detect the cell cycle based on previous research method [23]. In short, Huh-7 and HCCLM3 cell lines were subjected to treatment with transfection or β-Sitosterol with plasmids for 48 h, centrifuged, and re-suspended in pre-cooled PBS. Cell density was set to 2 × 10^5^ cells/mL and then fixed (4 ℃, overnight) in 1 mL 70% ethanol. Cells were centrifugated, and then re-suspended within pre-cooled PBS and then centrifuged again. The supernatant was removed. Next, cells were added with 500 µL PI staining solution (containing RNase), and slowly and thoroughly re-suspended. Cells were incubated (37 ℃, 1 h) in a warm water bath in a dark environment. After that, cells were placed on ice and detected using a flow cytometer (BD Biosciences, San Jose, USA) within 24 h.

The Annexin V-PI double staining kit (Nanjing Keygen Biotech CO., Ltd, Nanjing, China) was used to detect cell apoptosis. In brief, Huh-7 and HCCLM3 cell lines were seeded onto six-well plates at a density of 1 × 10^5^ cells per well and transfected with plasmids and treated with β-Sitosterol or CHIR-98014 for 48 h. Next, cells were collected and re-suspended in 500 μL of buffer. After that, 5 μL of Annexin V-FITC was added and mixed, followed by adding 5 μL of PI and mixing for reaction (room temperature, 15 min) away from light. A flow cytometer (BD Biosciences) was employed to analyze cell apoptosis immediately.

### Scratch assay

The scratch assay were applied to determine migration ability of Huh-7 and HCCLM3 cells [24]. Huh-7 and HCCLM3 cell lines were transfected for 48 h. Then, the cells were seeded into six-well plates an cultivated until they reached 90% confluence. A 200 μL pipette tip was used to scratch the cells. Followed ,by 3 times PBS washes, the cells were pretreated with mitomycin C for 1 h and receiving β-Sitosterol treatment in a humidified incubator (37 ℃, 5% CO_2_). After incubation, the cells were imaged at 0 and 48 h.

### Transwell assay

The Matrigel-coated Transwell chambers were used to measure invasion ability of Huh-7 and HCCLM3 cells according to previously described method [25]. Cells transfected with β-Sitosterol or CHIR-98014 treatments were placed in the top chamber, while the bottom chamber was filled with DMEM containing 10% FBS. The bottom surface of the upper chamber was stained with crystal violet dye for 20 min. Finally, a microscope was used to observe the number of invasive cells.

### Western blot

RIPA lysis buffer (Beyotime) was used to extract total protein from Huh-7 and HCCLM3 cells. Protein content was detected using a bicinchoninic acid (BCA) reagent kit (Beyotime). After separation by polyacrylamide gel electrophoresis, the protein was electroblotted onto nitrocellulose filter (NC) membranes (Merck Millipore, Billerica, USA). The membranes were then block with TBST solution containing 5% non-fat milk at room temperature for 2 h, followed by overnight incubation at 4 ℃ with primary antibodies including anti-BAX, BCL2, anti-cleaved caspase3, anti-total caspase3, anti-E-cadherin, anti-Snail, Anti-Vimentin and anti-GAPDH. Antibody dilution and resources are listed in Table S1. Membranes were washed three times with TBST solution before being incubated for 2 h at room temperature with horseradish peroxidase (HRP)-conjugated goat anti-rabbit IgG (1:2000; ab6721; Abcam, Cambridge, USA), followed by three times washes with TBST solution. GAPDH served as the internal reference. Color development was carried out using enhanced chemiluminescence (ECL) (New Cell & Molecular Biotech Co., Ltd., Suzhou, China) [26].

### Bioinformatics analysis

The drug target genes of β-Sitosterol were collected from PubChem (https://pubchem.ncbi.nlm.nih.gov/) [27]. The GSE112790 microarray containing 183 hepatocellular carcinoma specimens and 15 normal control specimens, was downloaded from the GEO database (https://www.ncbi.nlm.nih.gov/geo/). The GSK3B gene expression analysis was performed on this dataset using the R language limma package with *t* test analysis. The raw data of GSK3B gene expression in GSE112790 dateset are provided in the Supplementary Material_2. Using the Cancer Genome Atlas (TCGA) combined with Genotype Tissue Expression (GTEx) cohort, GSK3B expression was analyzed between tumors and corresponding normal tissues in 20 tumors, including TCGA-Liver Hepatocellular Carcinoma (TCGA-LIHC), via SangerBox online tool (http://sangerbox.com/). The Kaplan–Meier plot analysis based on SangerBox tool was applied to evaluate the prognostic significance of GSK3B in hepatocellular carcinoma patients. Using the auto select best cutoff algorithm on the website, hepatocellular carcinoma patients were divided into two cohorts (high vs. low expression). Then, the hazard ratio (HR) and *p* value were calculated. In addition, the receiver operating characteristic (ROC) curve based on SangerBox tool was constructed to evaluate the feasibility of using the GSK3B expression level to distinguish hepatocellular carcinoma patients from normal subjects.

### Statistical analysis

The cell experiments were performed for three biologic replicates. All data were presented as mean ± SD. The One-way analysis of variance (ANOVA) followed Tukey’s post hoc test was performed using GraphPad Prism 8.0 software. The significance level was set at *p* < 0.05.

## Results

### β-Sitosterol inhibited the viability and proliferation of hepatocellular carcinoma cells in vitro

**Fig. 1:**
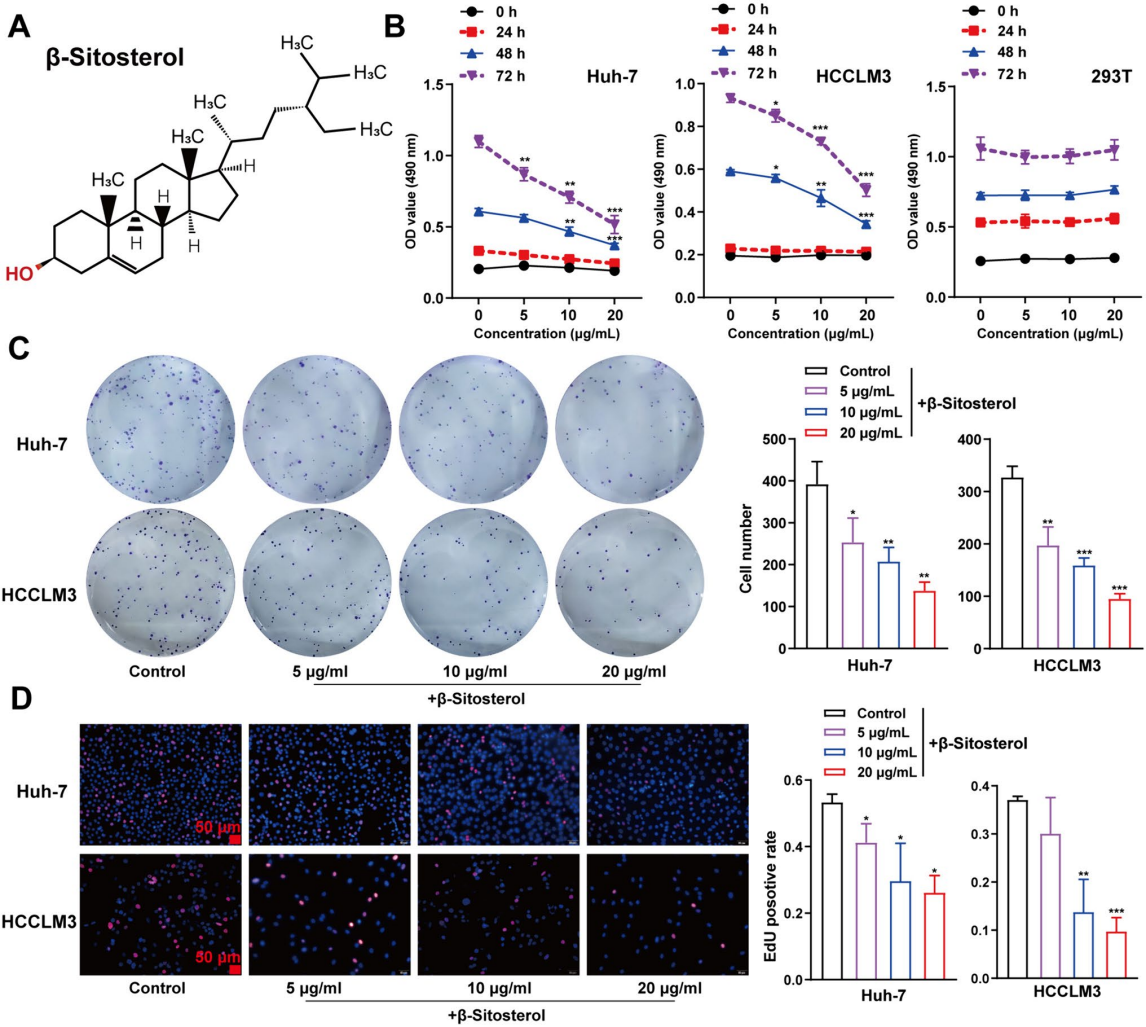
β-Sitosterol inhibited hepatocellular carcinoma cell viability and proliferation in vitro. **A** β-Sitosterol chemical structure. **B** After treating Huh-7, HCCLM3, and 293 T cells with β-Sitosterol of gradient concentrations (5 μg/mL, 10 μg/mL, and 20 μg/mL) for 24, 48, and 72 h, respectively; Huh-7, HCCLM3, and 239 T cell viability was detected using MTT assay. **C** Following a 48 h treatment of Huh-7 and HCCLM3 cells with β-Sitosterol (5 μg/mL, 10 μg/mL, and 20 μg/mL), Huh-7 and HCCLM3 cell proliferation was detected using (**C**) colony formation assay and (**D**) EdU assay. Scale bar = 50 µm. All *N* = 3 (biologic replicates). *p < 0.05, **p < 0.01, ***p < 0.001 compared to control group

The chemical structure of β-Sitosterol is displayed in Fig. 1A. To investigate the effect of β-Sitosterol on hepatocellular carcinoma cell viability, different concentrations (5 μg/mL, 10 μg/mL and 20 μg/mL) were used to treat Huh-7, HCCLM3, and 293 T cells for 24, 48, and 72 h. An MTT assay was performed to measure cell viability. Treatment with β-Sitosterol did not significantly affect the viability of 293 T cells but concentration-dependently decreased the activities of Huh-7 and HCCLM3 cells (Fig. 1B). After a 48 h treatment with β-Sitosterol at different concentrations (5 μg/mL, 10 μg/mL and 20 μg/mL), colony formation and EdU assays were conducted on Huh-7 and HCCLM3 cell lines. The results showed that β-Sitosterol treatment significantly reduced the proliferation of both cell lines in a concentration-dependent manner (Fig. 1C, D). Therefore, it can be concluded that β-Sitosterol suppress hepatocellular carcinoma cell viability and proliferation in vitro.

### β-Sitosterol promoted hepatocellular carcinoma cell apoptosis and affected cell cycle progression

**Fig. 2:**
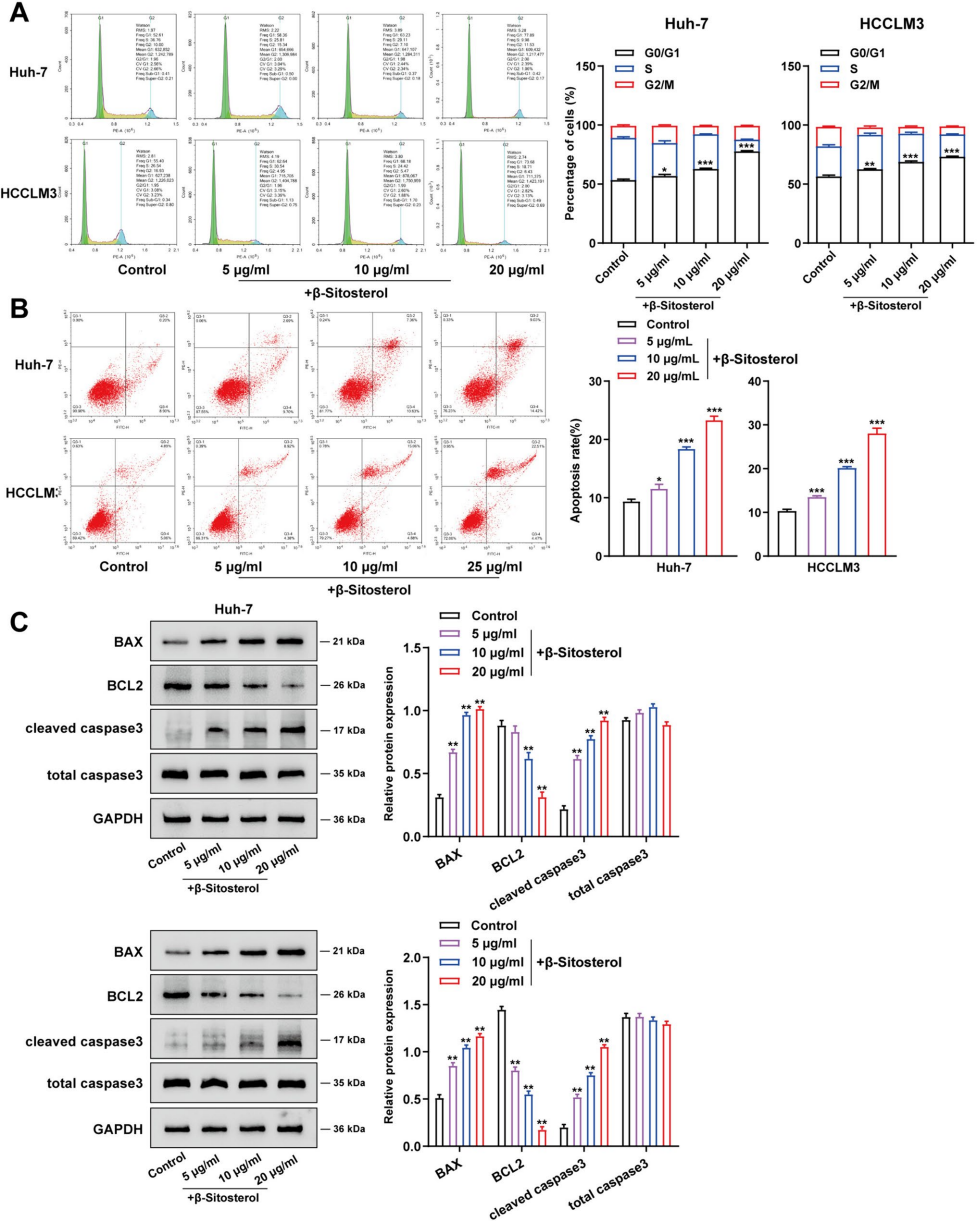
β-Sitosterol enhanced apoptosis and affected the cell cycle of hepatocellular carcinoma cells. After a 48 h treatment with β-Sitosterol (5 μg/mL, 10 μg/mL, and 20 μg/mL), flow cytometry was used to detect the (**A**) cell cycle and (**B**) apoptosis of Huh-7 and HCCLM3 cells; (**C**) BAX, BCL2, cleaved caspase3, and total caspase3 protein levels within Huh-7 and HCCLM3 cell lines were detected using Western blot. All *N* = 3 (biologic replicates). *p < 0.05, **p < 0.01, ***p < 0.001 compared to control group

The effect of β-Sitosterol on cell cycle and apoptosis of hepatocellular carcinoma cells was further explored. According to flow-cytometry results for cell cycle and apoptosis, β-Sitosterol induced G0/G1 phase arrest in the cell cycle and notably promoted Huh-7 and HCCLM3 cell apoptosis (Fig. 2A, B). In addition, Western blot was carried out to detect the level of apoptosis-associated proteins such as BAX, cleaved caspase3, BCL2, and total caspase3. Concentration-dependent treatment with β-Sitosterol has been shown to remarkably increase BAX and cleaved caspase3 protein levels while significantly reducing BCL2-protein levels in Huh-7 and HCCLM3 cell lines (Fig. 2C). In summary, β-Sitosterol can elicit G0/G1 phase arrest in the cell cycle and enhance hepatocellular carcinoma cell apoptosis.

### β-Sitosterol inhibited hepatocellular carcinoma cell migration, invasion, and EMT

**Fig. 3:**
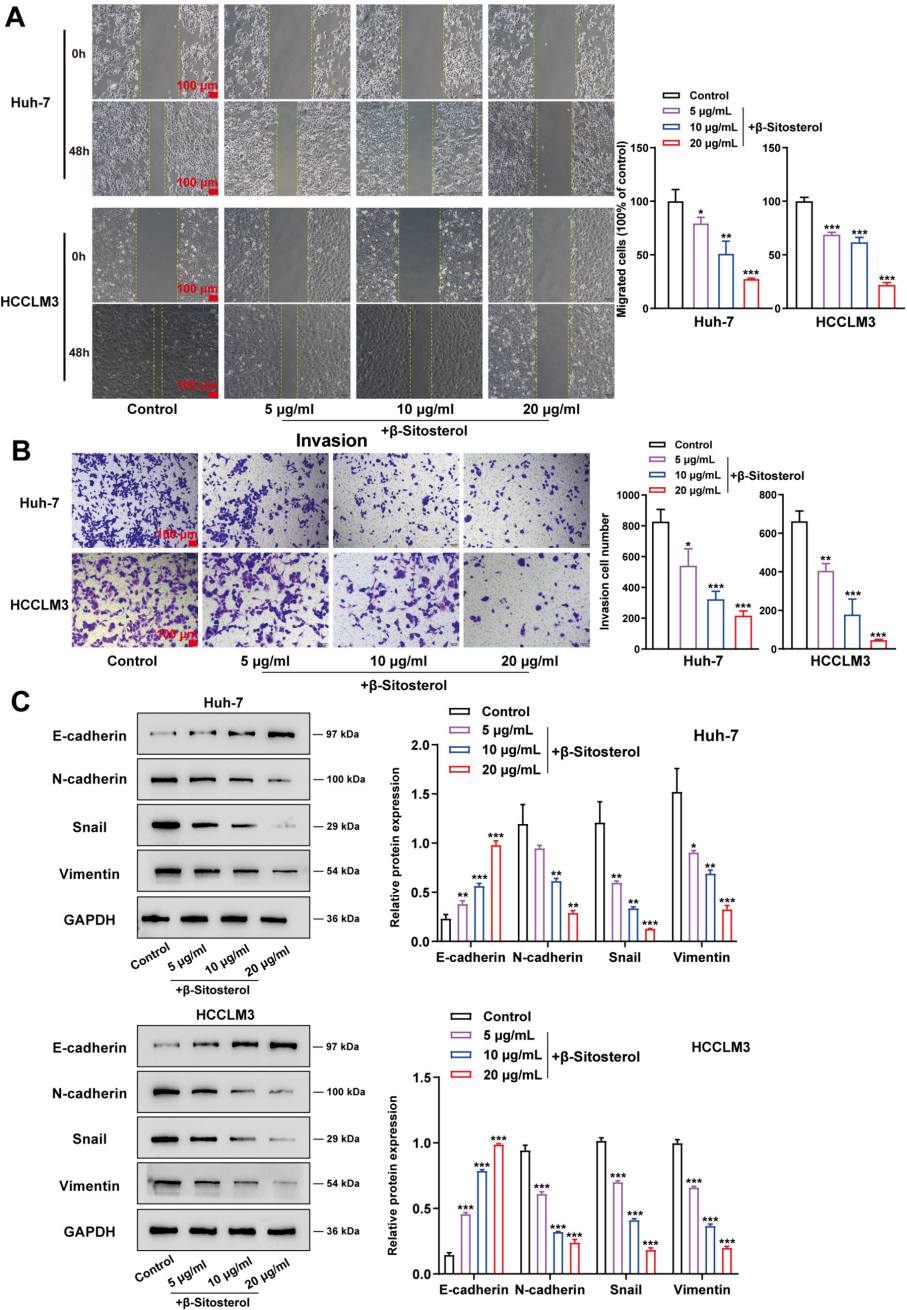
β-Sitosterol suppressed hepatocellular carcinoma cell migration, invasion, and EMT. After a 48 h treatment of Huh-7 and HCCLM3 cell lines with β-Sitosterol (5 μg/mL, 10 μg/mL, and 20 μg/mL), Huh-7 and HCCLM3 cell migratory ability was detected using (**A**) the scratch assay; (**B**) Huh-7 and HCCLM3 cell invasive ability was detected using Transwell assay; (**C**) E-cadherin, N-cadherin, Snail, and Vimentin protein levels within Huh-7 and HCCLM3 cell lines were detected using Western blot. Scale bar = 100 µm. All *N* = 3 (biologic replicates). *p < 0.05, **p < 0.01, ***p < 0.001 compared to control group

The effect of β-Sitosterol upon hepatocellular carcinoma cell migratory ability, invasion, and EMT was further explored. As shown in the results of scratch and Transwell assays, after β-Sitosterol treatment, the capacities of Huh-7 and HCCLM3 cells to migrate and invade were noticeably inhibited (Fig. 3A, B). Western blot analysis results revealed that β-Sitosterol promoted E-cadherin proteins whereas inhibiting N-cadherin, Snail, and Vimentin proteins in a dose-dependent manner in Huh-7 and HCCLM3 cell lines (Fig. 3C). Taken together, β-Sitosterol could suppress hepatocellular carcinoma cell migratory ability, invasive ability, and EMT.

### The target gene of β-Sitosterol was analyzed through network pharmacology

**Fig. 4:**
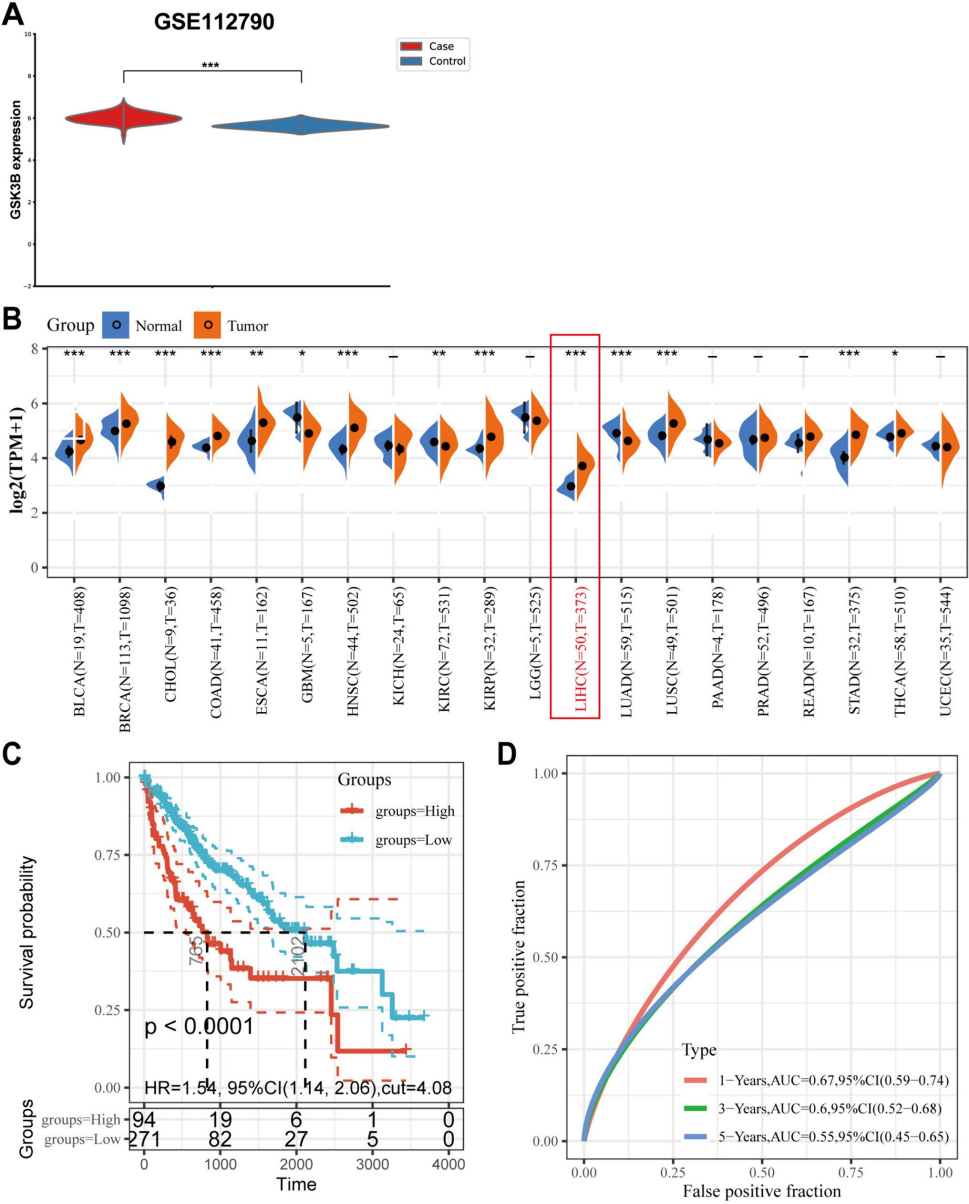
The target gene of β-Sitosterol was analyzed through network pharmacology. **A** GSK3B expression in dataset GSE112790 was identified by *t* test analysis. *** *p* < 0.001, vs. the control group. **B** GSK3B expression in the TCGA-LIHC database was identified. **C** Kaplan–Meier analysis was conducted to analyze the correlation between GSK3B and hepatocellular carcinoma patients’ prognosis and survival. **D** the accuracy of GSK3B in prognostic risk diagnosis for hepatocellular carcinoma was analyzed using the ROC curve

After screening through PubChem, GSK3B was identified as the only drug target gene of β-Sitosterol (Table S2). In addition, GSK3B expression was found to be upregulated in hepatocellular carcinoma tissues in GSE112790 dataset (Fig. 4A). Furthermore, SangerBox online analysis of the TCGA-LIHC database revealed that GSK3B was notably upregulated in hepatocellular carcinoma tissues (Fig. 4B). The survival analysis results indicated that a higher expression level of GSK3B in hepatocellular carcinoma patients could predict lower survival probability (Fig. 4C). According to the ROC curves, GSK3B exhibited good diagnostic potential for predicting the prognosis risk of hepatocellular carcinoma patients (Fig. 4D). From above all, these findings suggest that GSK3B functions as a potential drug target for β-Sitosterol in regulating biologic activities of hepatocellular carcinoma cells.

### Β-Sitosterol suppressed hepatocellular carcinoma cell proliferation and invasion and enhanced the apoptosis via inhibiting GSK3B expression

**Fig. 5:**
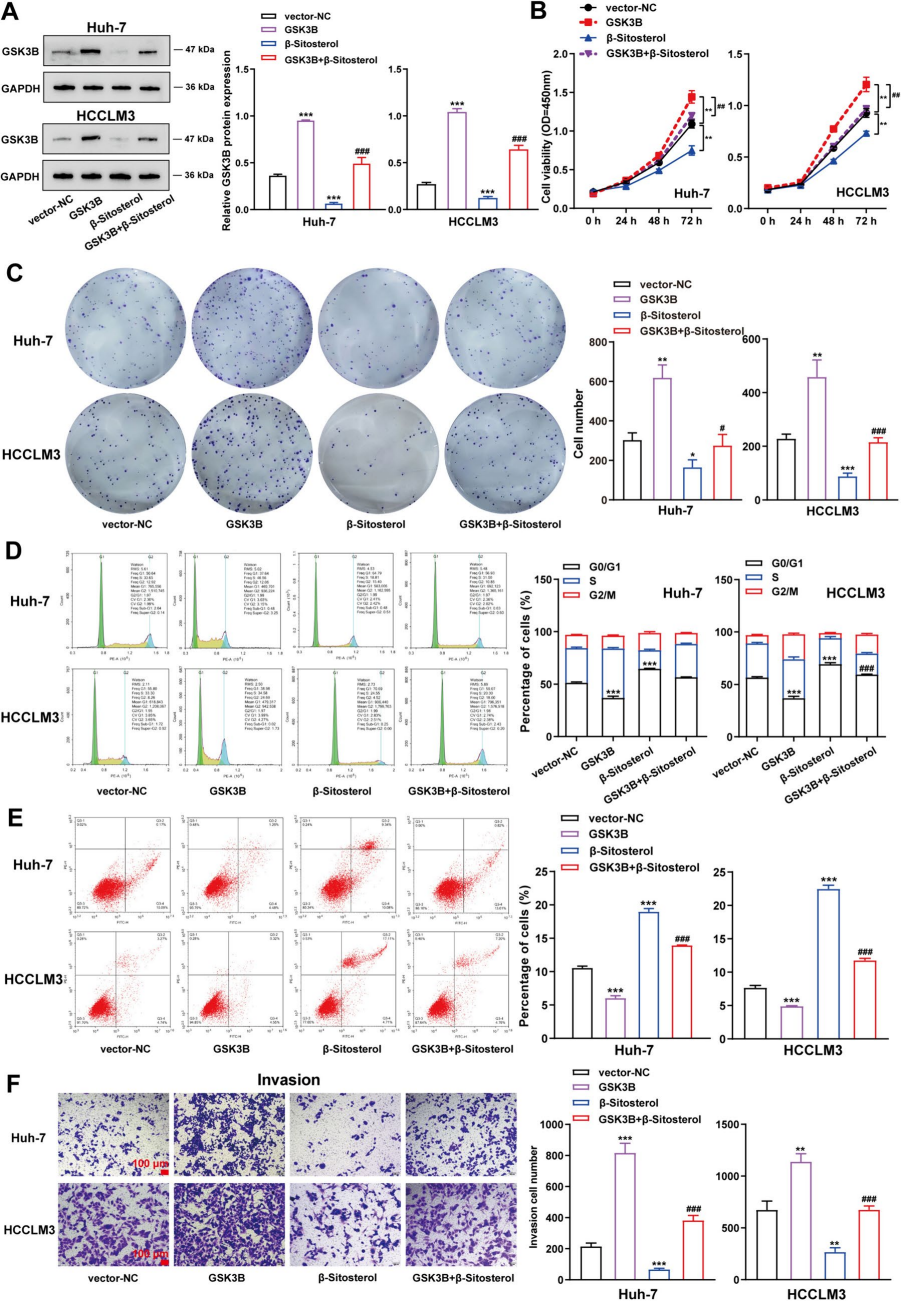
β-Sitosterol suppressed hepatocellular carcinoma cell proliferation and migration and enhanced the apoptosis via inhibiting GSK3B expression. After a 48 h transfection of Huh-7 and HCCLM3 cells with GSK3B overexpression vector, and then a 48 h treatment (24, 48 and 72 h treatment for CCK-8) with β-Sitosterol (10 μg/mL), (**A**) GSK3B-protein level within Huh-7 and HCCLM3 cell lines was detected using Western blot; (**B**) cell viability was detected using CCK-8 assay; (**C**) The proliferation of Huh-7 and HCCLM3 cells was detected using colony formation assay; the Huh-7 and HCCLM3 cell (**D**) cycle and (**E**) apoptosis were detected using flow cytometry; (**F**) Huh-7 and HCCLM3 cell invasion was detected using Transwell assay. Scale bar = 100 µm. All *N* = 3 (biologic replicates). *p < 0.05, **p < 0.01, ***p < 0.001 compared to the vector-NC group; #*p* < 0.05, ##*p* < 0.01, ###*p* < 0.001 compared to the β-Sitosterol group

To confirm that GSK3B was the target of β-Sitosterol, Huh-7, and HCCLM3 cell lines were transfected with the GSK3B overexpression vector and analyzed for GSK3B-protein levels using Western blot. The results showed that the GSK3B overexpression vector dramatically increased the amount of GSK3B protein in both cell lines (Fig. 5A). Cell proliferation was assessed by CCK-8 assay and colony formation assay, which revealed that GSK3B overexpression markedly promoted Huh-7 and HCCLM3 cell growth (Fig. 5B, C). Flow cytometry analysis demonstrated that GSK3B overexpression reduced the number of cells in the G0/G1 phase (Fig. 5D); while inhibiting apoptosis in both cell lines (Fig. 5E). Transwell assay indicated that GSK3B overexpression enhanced Huh-7 and HCCLM3 cell invasion (Fig. 5F). Furthermore, a series of experiments with β-Sitosterol were conducted, revealing that treatment with this compound inhibited GSK3B-protein content, suppressed cell proliferation and invasion, induced cell apoptosis, and caused arrest at the G0/G1 phase stage of the cell cycle in both Huh-7 and HCCLM3 cells; however, these effects could be partially reversed by GSK3B overexpression (Fig. 5A–F). In summary, our findings suggest that upregulation of endogenous or exogenous of GSK3B can counteract some but not all inhibitory effects exerted by β-Sitosterol on hepatocellular carcinoma cells.

### Comparison of the effects of β-Sitosterol and GSK-3 inhibitor on cell proliferation, invasion and apoptosis of hepatocellular carcinoma cells

**Fig. 6:**
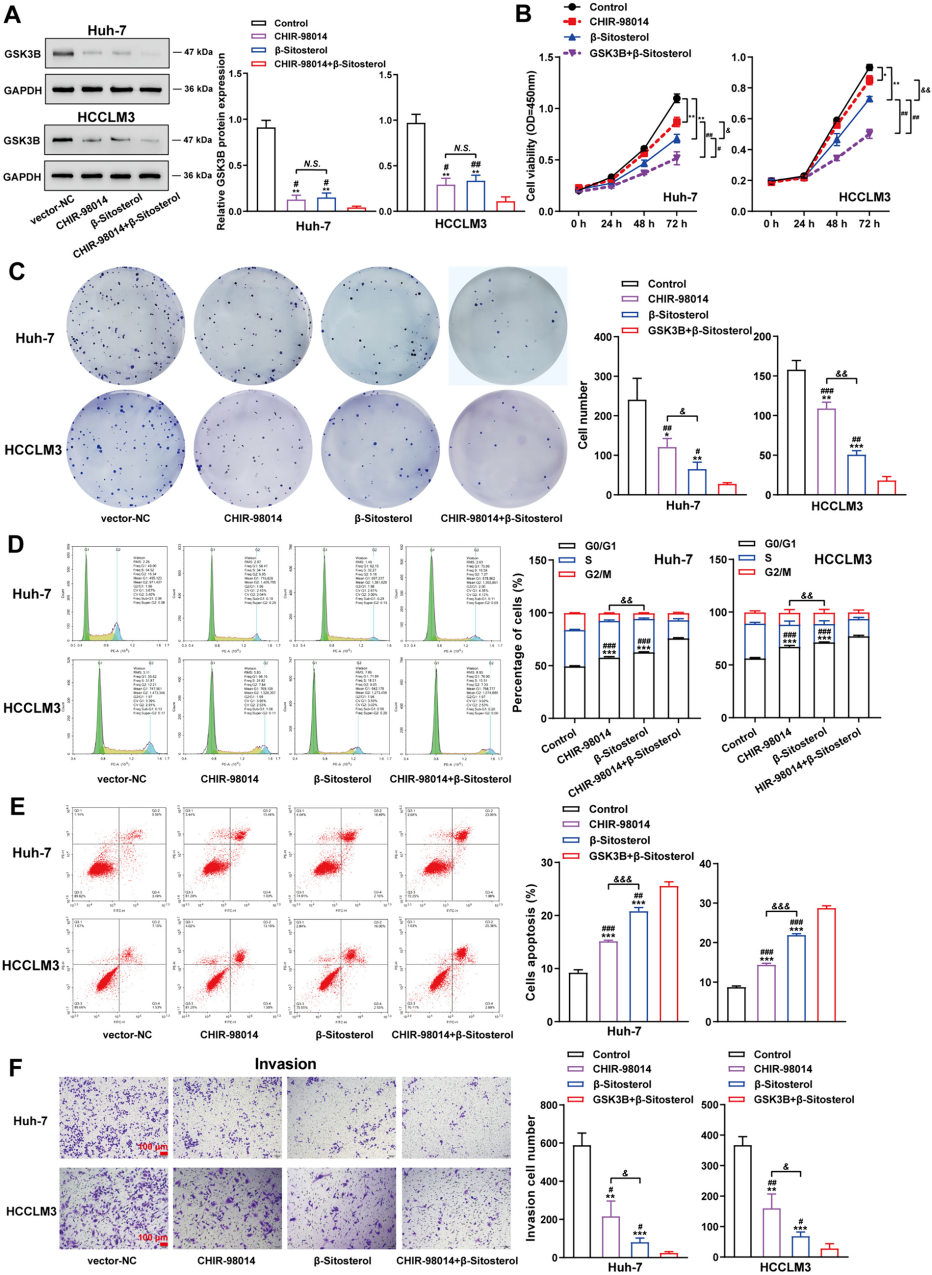
Comparison of the effects of β-Sitosterol and GSK-3 inhibitor on hepatocellular carcinoma cells. Huh-7, and HCCLM3 cells were treated with β-Sitosterol (10 μg/mL) and/or GSK3B inhibitor (50 nM CHIR-98014) for 48 h, and then examined for: (**A**) GSK3B-protein level by Western blot; (**B**) cell viability using CCK-8 assay; (**C**) cell proliferation using colony formation assay; (**D**–**E**) cell cycle and apoptosis using flow cytometry; (**F**) cell invasion using Transwell assay. Scale bar = 100 µm. All *N* = 3 (biologic replicates). *p < 0.05, **p < 0.01, ***p < 0.001 compared to the control group; #*p* < 0.05, ##*p* < 0.01, ###*p* < 0.001 compared to the CHIR-98014 + β-Sitosterol group. &*p* < 0.05, &&*p* < 0.01, &&&*p* < 0.001 compared to the CHIR-98014 group

Huh-7, and HCCLM3 cells were treated with β-Sitosterol and/or GSK3B inhibitor (CHIR-98014) and then analyzed for GSK3B-protein levels using Western blot. GSK3B-protein level was notably restrained by β-Sitosterol or CHIR-98014 treatment alone, and further inhibited by co-treatment of β-Sitosterol and CHIR-98014 (Fig. 6A). Cell viability and cell proliferation of Huh-7 and HCCLM3 cells were inhibited by β-Sitosterol or CHIR-98014 treatment alone, and further suppressed by co-treatment of β-Sitosterol and CHIR-98014 (Fig. 6B, C). The cell cycle in both Huh-7 and HCCLM3 cells were arrested at the G0/G1 phase stage by β-Sitosterol or CHIR-98014 treatment alone, and further affected by co-treatment of β-Sitosterol and CHIR-98014 (Fig. 6D). β-Sitosterol or CHIR-98014 treatment alone markedly promoted cell apoptosis, and co-treatment of β-Sitosterol and CHIR-98014 further amplified the effect to cell apoptosis (Fig. 6E). While Huh-7 and HCCLM3 cells invasion were suppressed by β-Sitosterol or CHIR-98014 treatment alone, and further restrained by co-treatment of β-Sitosterol and CHIR-98014 (Fig. 6F). It is worth mentioning that in this study, the inhibitory effects of β-Sitosterol on liver cancer cells were more obvious than that of GSK-3 inhibitor (CHIR-98014) (Fig. 6B–F).

## Discussion

Hepatocellular carcinoma is the sixth most prevalent malignancy and the second leading cause of cancer-related deaths worldwide [28]. Although Sorafenib is the first-line targeted therapy for advanced hepatocellular carcinoma, its clinical response rate is low, and patients can only benefit from it for a short period of time before developing drug resistance [29, 30]. The combination regimen of atezolizumab plus bevacizumab has been approved as the world’s first approved first-line immunotherapy for liver cancer, significantly extending patients’ survival and improving their quality of life compared to Sorafenib [31, 32]. β-Sitosterol is a natural compound that has been shown to inhibit hepatocellular carcinoma cell proliferation [14, 33]. In addition, β-Sitosterol also has multiple beneficial characteristics such as high efficiency and low toxicity [34], making it a potential candidate drug for hepatocellular carcinoma. Nevertheless, the regulation and the potential mechanism of β-Sitosterol in hepatocellular carcinoma cells remain unclear. In this study, we aim to explore how β-Sitosterol regulates biologic function in hepatocellular carcinoma cells and investigate its potential molecular mechanism using network pharmacology.

Firstly, we investigated the regulatory effects of β-Sitosterol on hepatocellular carcinoma cell proliferation and apoptosis. As previously reported, β-Sitosterol exhibits anti-cancer properties by suppressing cancer cell proliferation and induce apoptosis [35]. In hepatocellular carcinoma cells, β-Sitosterol induces apoptosis and activates caspase-3 and -9, demonstrating its cytotoxic activities [14]. In this study, we treated both hepatocellular carcinama cells and 293 T cells with β-Sitosterol. The MTT assay results revealed a significant inhibition of hepatocellular carcinoma cell viability by β-Sitosterol without affecting 293 T cell viability. This indicates that selective cytotoxicity of β-Sitosterol toward hepatocellular carcinoma cells while being non-toxic to normal cells. Furthermore, it inhibits proliferation, induces G0/G1 phase arrest in the cell cycle, enhance apoptosis in hepatocellular carcinoma cells; it also upregulates BAX and cleaved caspase3 protein levels while downregulating BCL2-protein levels. These findings demonstrated that β-Sitosterol can effectively suppress the growth of hepatocellular carcinoma cells through mechanisms involving inhibition of proliferation, induction of apoptosis and G0/G1 phase arrest in the cell cycle.

The excessive growth of cancer cells and distant metastasis of cancer cells are both contributing factors to the low survival rate in patients [36]. Cell migration, invasion, and EMT play crucial roles in the process of cancer cell distant metastasis [37]. Previous studies have demonstrated that β-Sitosterol can effectively suppress pancreatic cancer cell migration, invasion, and EMT when combined with gemcitabine, highlighting its anti-pancreatic cancer properties [38]. Therefore, this study aimed to investigated the inhibitory effects of β-Sitosterol on hepatocellular carcinoma cell migration, invasion, and EMT. The results indicate that β-Sitosterol significantly suppressed HCC cell migration and invasion while reducing N-cadherin, Snail, and Vimentin protein levels and increasing E-cadherin protein levels. Consistent with these findings, β-Sitosterol was also exhibited inhibitory effects on pancreatic cancer cell migration, invasion, and EMT [38]. Overall, the results suggest that β-Sitosterol exerts inhibitory effects on HCC cell migration, invasion, and EMT.

After investigating the inhibitory effects of β-Sitosterol on hepatocellular carcinoma cell growth, apoptosis, invasion, and EMT, we explored the underlying molecular mechanism. By analyzing PubChem online data, we identified GSK3B as the target gene for β-Sitosterol. Previous studies have demonstrated that GSK3B is a serine/threonine protein kinase [19, 39] and its inhibition can suppress cancer progression. For instance, an inhibitor of GSK3B has been found to decrease pancreatic cancer growth and metastasis in mice [40]. In breast cancer, inhibiting overexpression of GSK3B can also overcome chemoresistance [41]. Bioinformatics analysis revealed upregulation of GSK3B in hepatocellular carcinoma tissue and its potential as a diagnostic marker for poor prognosis in patients with this disease. Therefore, GSK3B represents a promising drug strategy for β-Sitosterol. GSK3B inhibitor (CHIR-98014) notably inhibited cell proliferation and invasion, promoted cell apoptosis and cell cycle arrest at G0/G1 phase in in hepatocellular carcinoma cells. β-Sitosterol treatment further promoted the efffects of GSK3B inhibitor on hepatocellular carcinoma cells. Moreover, our results from combined treatment (GSK3B overexpression and/or β-Sitosterol treatment) demonstrated that increased expression of GSK3B enhanced the proliferative and invasive capacity of cancer cells while reducing cell cycle arrest at G0/G1 phase and cell apoptosis; moreover, it partially counteracted inhibitory effects of β-Sitosterol on malignant behaviors in hepatocellular carcinoma cells. In conclusion, our findings support that targeting GSK3B is crucial for regulating biologic activities in hepatocellular carcinoma cells by using β-Sitosterol.

Nevertheless, the limitations of this study remain to be considered. Firstly, in or study, the in vivo effects of β-Sitosterol on hepatocellular carcinoma cells is still remains vague. In future studies, we may employ experiments in animal models such as mice or rats. Moreover, there could be other genes regulated by β-Sitosterol and involved in hepatocellular carcinoma advancement. The β-Sitosterol’s potent effects based on the GSK3B need further verify in hepatocellular carcinoma cell lines (Huh-7, HCCLM3) or liver cancer patient samples by transcriptomic analysis.

In conclusion, this study has validated the inhibitory effects of β-Sitosterol on the growth and metastasis of HCC cells in vitro; Through network pharmacology analysis, GSK3B has been identified as the target gene for β-Sitosterol. Subsequent experiments have comfirmed that the suppression of GSK3B expression mediates the anti-cancer properties of β-Sitosterol against HCC cell growth and metastasis. These findings provide a rationale for further investigation into the potential anti-cancer properties of β-Sitosterol through animal studies and clinical trials.

### Supplementary Information

Below is the link to the electronic supplementary material.

Supplementary file1 (DOCX 421 KB)

Supplementary file2 (XLSX 15 KB)

Supplementary file3 (DOCX 16 KB)

Supplementary file4 (DOCX 15 KB)

## Data Availability

All data supporting the findings of this study are available within the paper and its Supplementary Information.

## Abbreviations

BAX: BCL2 associated X
BCL2: B-cell lymphoma-2
CCK-8: Cell counting kit-8
EMT: Epithelial–mesenchymal transition
GSK3B: Glycogen synthase kinase 3 beta
HR: Hazard ratio
MTT: 3-(4:5-Dimethylthiazol-2-yl)-2:5-diphenyltetrazolium bromide
ROC: Receiver operating characteristic
TCM: Traditional Chinese medicine
TCGA-LIHC: The cancer genome atlas-liver hepatocellular carcinoma
