# Supplementary material for: β-Sitosterol alleviates the malignant phenotype of hepatocellular carcinoma cells via inhibiting GSK3B expression
Source: Hum Cell. 2024 May 30;37(4):1156–69. doi: 10.1007/s13577-024-01081-y (PMC11194219; doi:10.1007/s13577-024-01081-y)

**Supplementary Material_1. Identification of pLVX-GSK3B plasmid**

1. **Vector map of pLVX-GSK3B:**


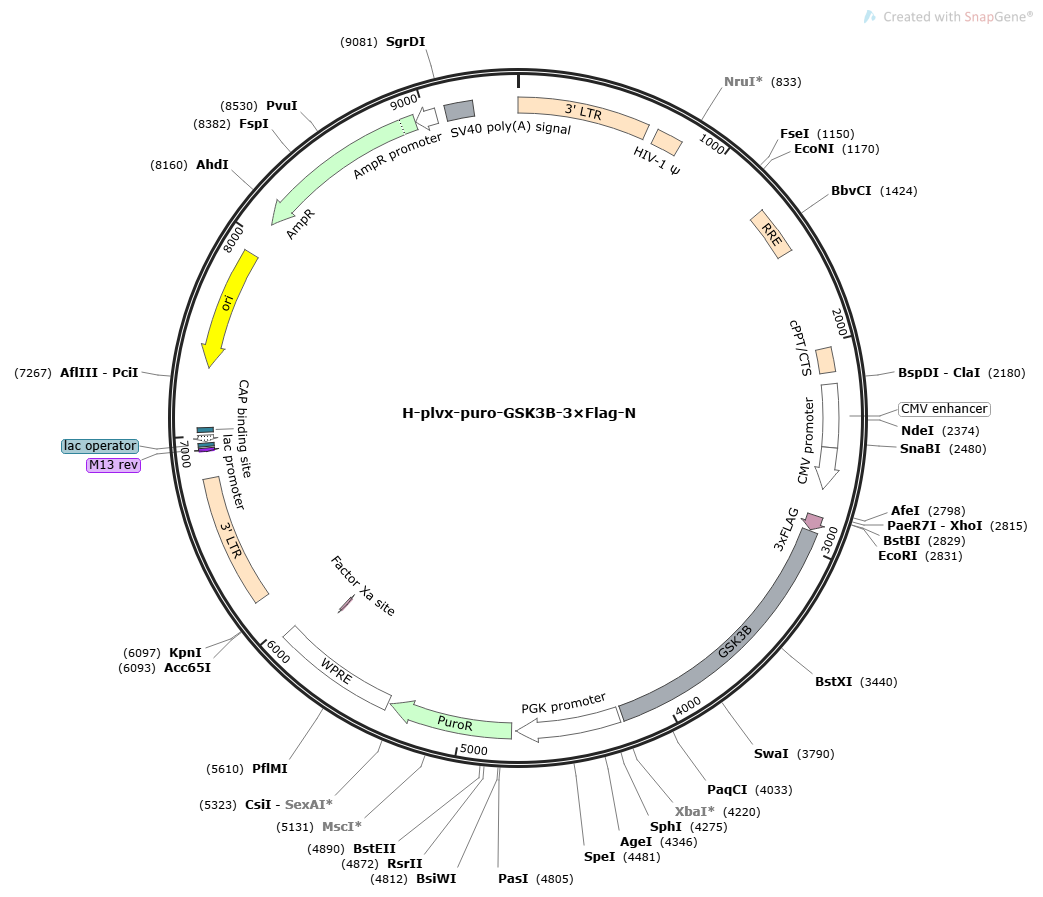


1. **Agarose gel electrophoresis of pLVX-GSK3B:**


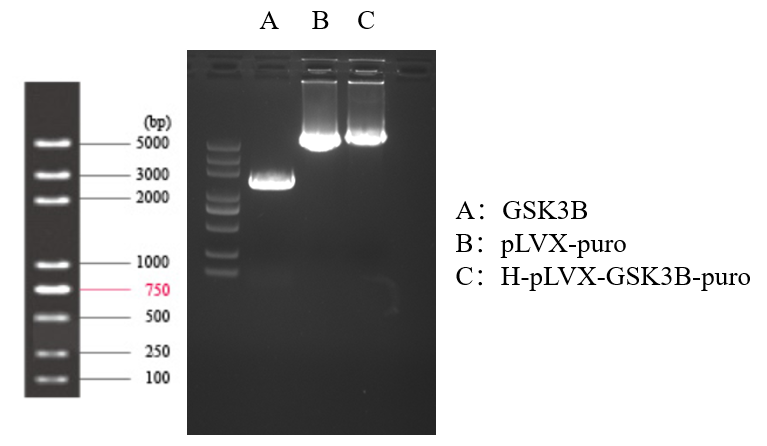


1. **Sequencing of pLVX-GSK3B：**

hGSK3B-L1302-Forward：

ATGATGACGATAAAGGATCCATGTCAGGGCGGCCCAGA


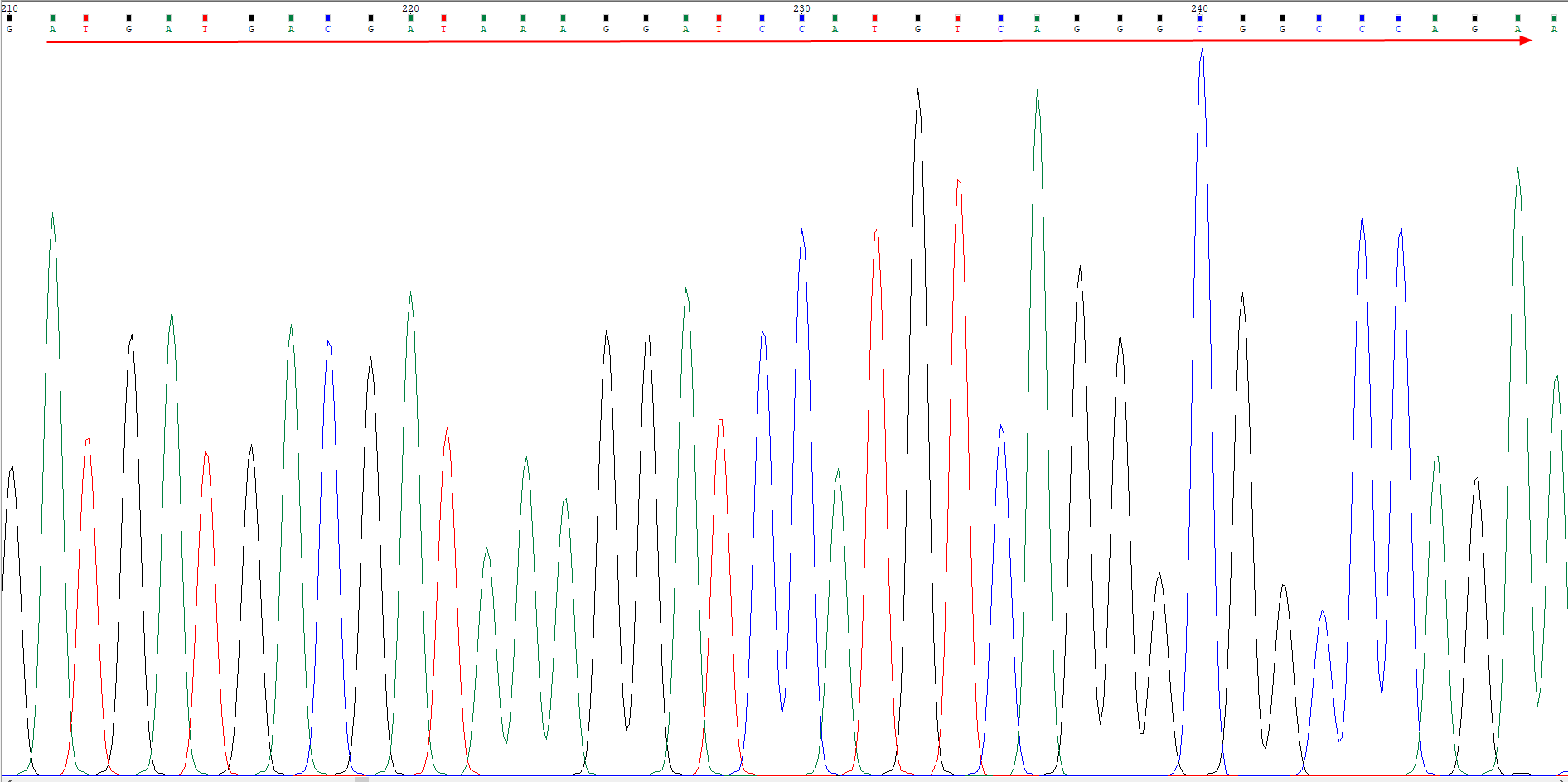

Supplement: Supplementary file 1 — Supplementary file1 (DOCX 421 KB) [file 13577_2024_1081_MOESM1_ESM.docx]
