# Supplementary material for: β-Sitosterol alleviates the malignant phenotype of hepatocellular carcinoma cells via inhibiting GSK3B expression
Source: Hum Cell. 2024 May 30;37(4):1156–69. doi: 10.1007/s13577-024-01081-y (PMC11194219; doi:10.1007/s13577-024-01081-y)
Supplement: Supplementary file 3 — Supplementary file3 (DOCX 16 KB) [file 13577_2024_1081_MOESM3_ESM.docx]

Table S1. Antibody information

| Name | Host species | Dilution | Cat. Number | Brand |
| --- | --- | --- | --- | --- |
| Anti-BAX | Rabbit | 1:1000 | ab32503 | Abcam |
| anti-BCL2 | Rabbit | 1:1000 | ab32124 | Abcam |
| anti-cleaved caspase3 | Rabbit | 1:500 | ab32042 | Abcam |
| anti-total caspase3 | Rabbit | 1:5000 | ab32351 | Abcam |
| anti-E-cadherin | Rabbit | 1:1000 | ab40772 | Abcam |
| anti-N-cadherin | Rabbit | 1:1000 | ab76011 | Abcam |
| anti-Snail | Rabbit | 1:5000 | ab216347 | Abcam |
| Anti-Vimentin | Rabbit | 1:1000 | ab92547 | Abcam |
| Ani-GAPDH | Rabbit | 1:2500 | ab9485 | Abcam |
