# Supplementary material for: β-Sitosterol alleviates the malignant phenotype of hepatocellular carcinoma cells via inhibiting GSK3B expression
Source: Hum Cell. 2024 May 30;37(4):1156–69. doi: 10.1007/s13577-024-01081-y (PMC11194219; doi:10.1007/s13577-024-01081-y)
Supplement: Supplementary file 4 — Supplementary file4 (DOCX 15 KB) [file 13577_2024_1081_MOESM4_ESM.docx]

Table S2. The drug target gene of β-Sitosterol through PubChem

| **Name** | **Mol ID** | **MW** | **Caco-2** | **OB(%)** | **DL** | **Target** |
| --- | --- | --- | --- | --- | --- | --- |
| β-sitosterol | MOL000358 | 414.79 | 1.32 | 36.91 | 0.75 | 1 |
